# Adipocire in the Fluid of a Hydrocele
*Dr. Bostock. Med.-Chir. Trans. Vol. XV.


**Published:** 1829-10-01

**Authors:** 


### XLIII.

### ADIPOCIRE IN THE FLUID OF A HYDROCELE.*

Those who have witnessed many operations for hydrocele, must sometimes have observed small scales of white spermaceti-looking matter floating in the fluid drawn off. Not very long ago, a deservedly eminent surgeon in this town being curious to know what the substance in question might be, sent the fluid in which it was contained to a distinguished chemist, requesting his opinion on its nature. To his no small surprise there came back, in the course of a few days, a bottle, containing some very pretty crystals, and a note, importing that they were adipocire, the product of the chemical examination of what had been sent! The worst of it is that the chemists, like the microscopists, have it all their own way. Nobody can see as far as the one, nobody can possibly contradict the other. When a scientific gentleman transmutes some dirty-looking water into brilliant crystals, what can we do but admire and believe? Seriously, however, it is a curious circumstance that the living body should present, as a product of disease, what used to be considered as a change exclusively produced by the gradual operation of certain decomposing agents after death. The fluid which was the subject of the present analysis was put into Dr. Bostock's hands by Mr. Earle, who had drawn it off from a hydrocele of some years' standing. It was of reddish-brown colour and of viscid consistence, with some masses of mucus floating in it, while some pearly scales of whitish matter lay at

* Dr. Bostock. Med.-Chir. Trans. Vol. XV.

the bottom of the vessel. On agitation, these scales were diffused through the fluid, on which they conferred a shining and glossy appearance.

"I immediately recognized the fluid as resembling some of which we have a notice in the early volumes of our Transactions, the first by Dr. Marcet, and the others by myself; the former obtained from a diseased thyroid gland, the latter from tumours seated in the muscular parts. Dr. Marcet ascertained that these white particles were inflammable and partially soluble in alcohol; my observations, in a great measure, coincided with his, and led me to conclude, that it was a substance, intermediate, in its chemical properties, between albumen and wax; I therefore named it albumino-cerous matter. A substance of the same appearance has been since detected by M. Breschet and by Dr. Christison in various morbid fluids, which in its chemical relations was found to resemble cholesterine; so that this white scaly matter appears to be occasionally formed in various parts of the body, and in connexion with various structures. But as the above substances, although agreeing in their leading properties, both physical and chemical, differed in some minor points, I thought the matter in question deserving of a more detailed examination."

Dr. B. accordingly instituted a series of experiments, which those who are curious respecting the subject may find in the original paper. We cannot afford the space for their insertion, but must be content to acquaint our readers with the conclusions to which they led the able experimentalist.

"The above experiments seem to prove, that the white scaly matter which was formed in the fluid in question, was not precisely similar to any of the proximate principles generally recognized; it was essentially different from albumen, from adipose matter, from osmazome, from adipocire, and from cholesterine. It may be briefly characterized as a substance disposed to assume the form of thin plates or scales of a white colour and pearly lustre, of a somewhat unctuous consistence, fusible at a high temperature, inflammable, and burning with a bright flame. It is not soluble in water or in alcohol, nor is it capable of being saponified by potash, but is partially soluble in ether.

"It would appear that a substance of the same *general* nature with the white scales described above is occasionally found connected with the solids, as well as with the fluids of the body. Besides its existence as the principal constituent in the biliary calculi, it seems to be not unfrequently found in the substance of the liver, in certain diseased states of this viscus, as well as in tumours and morbid depositions of various kinds. We may conjecture that this peculiar substance is immediately derived from the albumen of the blood, and we may further conjecture that it is an extra-vascular production, that it does not exist ready formed in the blood, or any of its component parts, as long as they are under the influence of the circulation. It would appear probable, that when an albuminous secretion is deposited in a cavity, and retained there for a certain length of time, a part of its animal matter is converted into this substance. We have not indeed been able hitherto to form it out of the body by any artificial means, but its nature and the situations in which it is found favour this supposition. The process we may regard as analogous to that by which the muscular fibre is converted into adipocire,* the substances produced bearing a certain relation to each other, and the principal agents in both cases, being certain states of moisture and temperature. As it is always convenient to have a specific name for every substance which possesses specific properties, I propose

* "An opportunity lately occurred to me of proving decidedly, that adipocire is the immediate production of the muscular fibre, not, as has been supposed by some eminent chemists, a mere residue of the fat, after the destruction of the muscle. I obtained a portion of a human thigh, which, in consequence of long maceration in water, had undergone the change in question, the form to retain the appellation, which I formerly suggested, of albumino-cerous matter, which may at least be employed as a temporary or provisional term, until we have one that is more appropriate.

"According to this view of the subject, the substance in question must be regarded, rather as the cause than the consequence of disease; the primary morbid change not being in the fluid, but in the organ into which it is deposited; the substance, after its deposition, giving rise to further derangement, either by unduly distending the parts, or by mechanically obstructing the vessels. We possess no agent by which the substance, when once deposited, can be removed, nor are we acquainted with any means of preventing its formation. Fortunately, however, in most cases, the situation and the mode of its deposition place it out of the reach of the more active and vital parts of the system, and render it rather an object of physiological curiosity than of medical treatment."

of the muscles was preserved, and there was no apparent loss of substance; there was even an indistinct appearance of the fibrous texture. On heating portions of this substance with alcohol and with ether, the whole was dissolved, except the cellular web, which was left exhibiting its ordinary structure, and forming one-twelfth part only of the whole. An observation of nearly a similar kind had been previously made by Dr. Thomson; Ann. Phil. XII. 41."

